# Is shared decision-making vanishing at the end-of-life? A descriptive and qualitative study of advanced cancer patients’ involvement in specific therapies decision-making

**DOI:** 10.1186/s12904-015-0057-4

**Published:** 2015-11-16

**Authors:** Yvan Beaussant, Florence Mathieu-Nicot, Lionel Pazart, Christophe Tournigand, Serge Daneault, Elodie Cretin, Aurélie Godard-Marceau, Aline Chassagne, Hélène Trimaille, Carole Bouleuc, Patrice Cuynet, Eric Deconinck, Régis Aubry

**Affiliations:** 1 Department of Pain Management – Palliative Care, Besancon University Hospital, 2 Bd Fleming, 25000 Besancon, France; 2 Inserm CIT808, Besancon University Hospital, 2 place St-Jacques, 25000 Besancon, France; 3 Ethics Centre of Burgundy and Franche-Comté, Besancon University Hospital, 2 place St-Jacques, 25000 Besancon, France; 4 Department of Psychology of Besancon, University of Franche-Comté, 2 place St-Jacques, 25000 Besancon, France; 5 Department of Oncology, Henri Mondor University Hospital, 51, avenue du Mal de Lattre de Tassigny 94010, Créteil cedex, France; 6 Palliative Care Unit, Notre Dame Hospital, Montréal University Hospital (CHUM), Montreal, Canada; 7 Inter-Disciplinary Supportive Care Department for the Oncology Patient, Institut Curie, 26 Rue d’Ulm, 75005 Paris, France; 8 Besancon University Hospital, Hematology, 2 Bd Fleming, 25000 Besancon, France

**Keywords:** Neoplasms, Decision making, Uncertainty, Ethics, Physician-patient relations, Defense mecanisms, Withholding treatment, Advance care planning, Chemotherapy, Palliative care

## Abstract

**Background:**

Little is known about what is at stake at a subjective level for the oncologists and the advanced cancer patients when they face the question whether to continue, limit or stop specific therapies. We studied (1) the frequency of such questioning, and (2) subjective determinants of the decision-making process from the physicians’ and the patients’ perspectives.

**Methods:**

(1) All hospitalized patients were screened during 1 week in oncology and/or hematology units of five institutions. We included those with advanced cancer for whom a questioning about the pursuit, the limitation or the withholding of specific therapies (QST) was raised. (2) Qualitative design was based on in-depth interviews.

**Results:**

In conventional units, 12.8 % of cancer patients (26 out of 202) were concerned by a QST during the study period. Interviews were conducted with all physicians and 21 advanced cancer patients. The timing of this questioning occurred most frequently as physicians estimated life expectancy between 15 days and 3 months. Faced with the most frequent dilemma (uncertain risk-benefit balance), physicians showed different ways of involving patients. The first two were called the “no choice” models: 1) trying to resolve the dilemma via a technical answer or a “wait-and-see” posture, instead of involving the patients in the questioning and the thinking; and 2), giving a “last minute” choice to the patients, leaving to them the responsibility of the decision. In a third model, they engaged early in shared reflections and dialogue about uncertainties and limits with patients, proxies and care teams. These schematic trends influenced patients’ attitudes towards uncertainty and limits, as they were influenced by these ones. Individual and systemic barriers to a shared questioning were pointed out by physicians and patients.

**Conclusions:**

This study indicate to what extent these difficult decisions are related to physicians’ and patients’ respective and mutually influenced abilities to deal with and share about uncertainties and limits, throughout the disease trajectory. These insights may help physicians, patients and policy makers to enrich their understanding of underestimated and sensitive key issues of the decision-making process.

## Background

Cancer is the main cause of death in economically developed countries and the second in developing countries [1, 2]. The length and quality of life of these patients has considerably improved in the last years, thanks to new therapies and improvements in supportive care [3–5]. However, when the disease progresses and becomes refractory to treatments, physicians and patients face the question of whether to pursue, limit or withhold anti-cancer therapies [6].

Specific therapies (ST) used to control the evolution of advanced cancers are generally chemotherapies, and less frequently radiotherapies. When validated therapies fail to control the disease, it becomes unlikely that further ST will have superior effectiveness than uniquely supportive care [7–9]. Conversely, it has been shown that early palliative care in advanced pulmonary cancers is associated with an increased survival and quality of life [10, 11]. Fewer of these patients received chemotherapies in the last weeks of life [8]. In other advanced cancer, recent findings also support early palliative care [12]. Currently, the prescription of chemotherapies in the last weeks of life tends to be considered a negative outcome for the quality of end-of-life care. It is associated with increased emergency room admissions and hospital deaths, as well as to lower access to palliative care services [9, 13]. However, several studies show that the use of chemotherapies in the last weeks of life in advanced cancer patients is widespread [9, 13, 14] and increasing [15]. On average, these retrospective studies indicate that about 20 % of cancer patients had received chemotherapy during their last month of life.

It is likely that this paradox reflects the complexity of the decision to pursue, limit or stop ST in these situations. Loss of hope or sense of abandonment that patients may experience when ST have to be stopped are likely to interfere with medical or institutional determinants [11, 16, 17]. However, studies conducted to understand factors underlying such decisions remain rare, and, while highlighting the importance of subtle interactions between physicians and patients, they generally report only the physicians’ (and sometimes the nurses’) points of view [14, 18–22]. Furthermore, these studies mainly focus on the decision itself and less frequently on the prior questionings and deliberating process. To our knowledge, the questioning about the pursuit, the limitation or the withholding of ST (QST), from the compared physicians’ and the patients’ perspectives, has not yet been prospectively studied.

We present the results of a study exploring (1) the frequency of the questioning about the pursuit, the limitation or the withholding of ST in advanced cancer patients, and (2) the determinants and modalities of these questionings from the compared physicians’ and the patients’ perspectives, with a focus on the patients’ involvement.

## Methods

We conducted a prospective multi-centre observational study in five various institutions located in Paris and East of France: two university hospitals (one oncology unit in Paris and one hematology unit in Besançon), one general hospital (oncology / hematology unit of Colmar), one cancer centre (Oncology, in Nancy) and one private hospital (oncology, in Dijon). Patients were included from January to May 2011. Inclusion criteria were the following: adult patients, with advanced cancer or hematological malignancy, hospitalized during the time of the study, for whom the referent physician was questioning the pursuit, the limitation or the withdrawal of ST at the time of the study or had such a questioning within the two weeks preceding the investigation. The study was approved by the regional independent ethics committee (Comité de Protection des Personnes Grand Est II) under the number 10/580.

### Epidemiologic approach

The study included two different stages. First, an epidemiological and descriptive stage, in which we screened all hospitalized patients, during a given week in each centre, and identified and characterized those who matched the inclusion criteria. Department characteristics and institutional organization of the decision were collected using a first questionnaire. For eligible patients, patient characteristics and individual organization of the decision were anonymously collected using a second questionnaire. Data collected with these questionnaires for the present analysis are shown in Tables 1 and 2. The only exclusion criteria at this stage were pre-existing guidelines or research protocols establishing therapeutic action. For practical reasons, outpatients were not included. Statistical descriptive analysis was performed by the Clinical Investigation Centre (CIC) of Besançon, by the mean of the SAS software for Windows, version 9.1 (SAS Institut, Inc., Cary, NC).

**Table 1:** Distribution of the questionings about further specific therapies among the different centres

Investigation centre	Speciality	Number of beds			Frequency of the QST: nb of included patients/nb of hospitalized patients during the inclusion period (%)			Nb of patients interviewed
		Conventional	Ambulatory^a^	Total	Conventional	Ambulatory^a^	Total	
University Hospital	Haematology	39	14	53	4 / 41 (9.7)	1 / 99 (1)	5 / 140 (3.5)	5
Cancer centre	Medical Oncology	46	52	98	3 / 62 (4.8)	0 / 247 (0)	3 / 309 (0.9)	1
University Hospital	Medical Oncology	23	27	50	7 / 38 (18.4)	1 / 136 (0.7)	8 / 174 (4.5)	5
Private clinic	Medical Oncology-RT	31	19	50	6 / 28 (21.4)	0 / 22 (0)	6 / 50 (12)	5
General Hospital	Onco-haematology	23	35	58	6 / 33 (18.2)	1 / 133 (0.7)	7 / 166 (4.2)	5
		162	141	303	26 / 202 (12.8)	3 / 637 (0.4)	29 / 839 (3.4)	21

*QST* questioning about specific therapies, *nb* number, *RT* radiotherapy

^a^Ambulatory includes day and week hospitalization

**Table 2:** Patient characteristics

	n	Value	%
Age, years	29		
Mean		65,4	
Range		43–80	
Sex	29		
Male		12	41,3
Female		17	58,7
Primary tumor			
GI (+pancreas)		6	20,7
Breast		5	17,2
Respiratory + ENT		4	13,8
Other solid tumor		5	17,2
Leukemia		5	17,2
Lymphoma		3	10,3
Myeloma		1	3,4
Time from diagnosis, months	29		
Mean		44	
Range		0.8–186.4	
Performance status	28		
Mean		2,4	
Range		0–4	
0–1		5	17,2
2		6	20,7
3		14	28,0
4		3	10,3
Number of treatment lines	28		
Mean		2.8	
Range		0–6	
0		2	6,8
1		3	10,3
2		9	31,0
3		5	17,2
4		5	17,2
>4		5	17,2
Quality of life^a^	29		
Mean		2,9	
Range		0,2–9	
0–2,4		16	
2,5–4,9		8	
5–7,4		3	
7,5–10		2	
Estimated Life expectancy	29		
<15 days		1	
15 days–3 months		10	
3–6 months		8	
6–12 months		5	
Not answered		5	

^a^estimated between 0 and 10 on a visual scale by physician

### Qualitative analysis

The second stage aimed to understand the determinants and modalities of the questioning through semi-directed interviews with physicians and patients. Qualitative methodology was selected in order to access information on physicians’ and patients’ experiences which could not be obtained through a quantitative design, as well as to compare their points of view. Eligible patients were informed of the study and their signed consent was asked. Exclusion criteria concerned patients unable to be interviewed (poor general condition, patient discharged at time of interview), and patients perceived by the referent physician as psychologically unable to handle the interview. Interviews with the referent physician directly approached the determinants and modalities of their QST, whereas with patients, this specific questioning was used only if they were explicitly aware of it (semi-directive topic lists are shown in annex 1 and 2, respectively). If not, patients were interviewed about their general perception of the ST decision process, their involvement in the decisions, and the influence of their relationship with physicians, team and proxies on decisions.

YB (physician) interviewed the majority of the physicians, and FMN (psychologist) the majority of the patients. For some of the interviews these roles were reversed, in order to compare the two approaches. Mean duration of the interviews was 30 and 60 min for the physicians and the patients respectively. All the interviews were audiotaped and anonymously transcribed, taking into account non-verbal expression of emotions. We conducted qualitative analysis according to the grounded theory model [23]. Verbatim transcribed from the interviews were thematically analyzed, using Atlas.ti 6.2 software. Generated codes led to defined specific categories, and then conceptual theory explaining interactions between physicians and patients who were questioned. The main two researchers confronted their respective coding throughout the process of analysis in order to achieve constant consensus and to ensure accuracy of emerging categories. We chose to conduct interviews with all the physicians, and with the maximum of patients included in the epidemiological section of the study. The saturation of concepts was applied and eventually reached. Definitions of emerging codes and themes were written with typical examples from the interviews to ensure clarity in communicating meaning. We used Beauchamp and Childress’ definitions of ethical principles to describe value conflicts faced by physicians [24]. We addressed these recognized criteria for qualitative research: credibility, concordance, auditability, and confirmability [25]. Credibility was assessed by regular debriefing of the data collectors with a multidisciplinary team drawing expertise from health sciences, sociology, psychology, philosophy, and ethics, and by independent coding and analysis by the investigators. Concordance was assessed through line-by-line analysis of the interview transcripts and by providing extensive examples of the data. Detailed coding and memos written throughout the analysis enhanced auditability, enabling an examination of the “decision trail” used. Consistency of the investigators’ independent coding was examined and confirmed by the research team. A professional translator translated the quotes that we eventually chose to illustrate our results.

## Results

### Descriptive results

#### Frequency of the questioning

Among 839 cancer patients hospitalized in the five centres during the inclusion week, 29 were concerned by the QST of their physician (3.4 %). In conventional and intensive care units, excluding day and week hospitalization units, this proportion reached 12.8 % (26 out of 202). Table 1 presents the distribution of patients concerned by the questioning according to their investigation centre and the type of unit they were hospitalized in. Among the 29 patients identified, we could conduct all the interviews with the referent physicians (*n* = 17) and 21 with the patients. All participants (both physicians and patients) gave signed informed consent before taking part. Reasons why eight patients were not interviewed are the following: three were too physically ill, three had been discharged at the time of the interviews, one did not speak French, finally, a physician decided to exclude one of his patients because she was considered too anxious and to be maintaining an unrealistic hope for cure. Five physicians were interviewed two times, two physicians were interviewed three times, and one physician was interviewed four times.

#### Patient characteristics

Patients included were 12 men and 17 women, with a median age of 68 (range 43–80). Time from diagnosis to inclusion date was 3.6 years (range 0.8–186.4 months). Twenty patients were receiving ST at time of the inclusion. For seven other patients, ST had been withheld for a mean time of 3.5 months (range 1–13). The last two patients had never received any ST. Characteristics of the diseases and the clinical situations are presented in Table 2.

### Qualitative findings

Three kind of uncertainty led the interviewed physicians to a QST: 1) an uncertain risk-benefit balance; 2) a discrepancy between therapeutic possibilities and patients’ preferences, i.e. unfavorable risk-benefit balance whereas the patient wished to continue treatments; 3) the opposite discrepancy between therapeutic possibilities and patients’ preferences, i.e. favorable risk-benefit balance whereas the patient was unwilling to continue treatment.

Facing the most frequent dilemma (uncertain risk-benefit balance), physicians showed two predominant attitudes towards patients, that we named the “no choice” models. The first attitude was trying to resolve the dilemma via a technical answer or a “wait-and-see” posture, instead of involving them in their questioning and reflection (theme A). The second attitude was giving a “last minute” choice to the patient (theme B). Rarer, physicians described an early engagement in shared reflections and dialogue about uncertainties and limits with patients, proxies and teams (theme C).

**Theme A – First “no choice” model: trying to resolve the dilemma with a technical answer or a “wait-and-see” posture, instead of involving the patients in their questioning.**

Most physicians, before seeking to inform patients about their uncertainty and involving them in the decision, waited to resolve the dilemma between beneficence and non-maleficence by either finding a technical answer that temporally removed doubts, or waiting for the evolution of the situation to impose an obvious decision.

*He’s a patient who has high expectations from treatments… We never brought it up [our doubts] with him; neither had we ever sought to question such things… No, we never brought it up directly with him. Upon entering his room, the first thing he said was: “See… It [tumour localized on the face] is growing! Can we do something?” This was his question. And as we had discussed it just before going into his room, we could answer immediately: “Yes, we can try something.”* **Physician 4, hematologist.**

Based on physicians’ own words, it seemed that the attitude of not addressing the issue of uncertainty with patients was frequently the rule earlier in the disease trajectory, when uncertainty between benefit and risk was less crucial.

*YB: Have you ever discuss the current situation of uncertainty with the patient? MD: Not yet, because we still are in a dynamic of treatments. Current progression of the disease, while we were coming from a cycle of chemotherapy… let’s say that this issue hasn’t yet been addressed.* **Physician 27, oncologist.**

Thus, the issue of uncertainty frequently emerged in the discussions between physicians and patients as physicians realized that pursuing ST would no longer be an option because ST and/or the patients were exhausted. The decision to stop ST was described as difficult when exhaustion of ST and exhaustion of the patient wasn’t simultaneous. This was the case in the second and the third kind of uncertainty described above. These situations could correspond to a lack of questioning from the physicians themselves about the relevance of ST in advanced cancer, especially earlier in the disease trajectory.

*YB: During the follow-up, did you discuss with this patient the fact that chemotherapy should be stopped at a certain point? MD: No, because until the third line… It’s only realizing that the third line [of chemotherapy] didn’t work, that we told ourselves that it wasn’t reasonable to continue. YB: So, it wasn’t discussed… Did the patient ever asked questions about that? MD: No. YB: Does it happen that patients sometimes ask about the possibility that treatments should be stopped? MD: Rarely… They’re always waiting for the next treatment.* **Physician 8, oncologist.**

Decisive action, rather than an involvement in a physician’s possible questioning, was also explicitly or implicitly expected by the majority of patients.

*Personally, I have always told the doctor: “it’s you who decide, not me”. He’s the one who decides for my treatment, it’s not me. He’s the one who knows. And that’s it! I trust him; he’s a very good doctor. I trust him totally!* **Patient 12.**

*YB: Were you able to talk with the team or the doctor about these uncertainties, these issues you’re worried about? P: I don’t want to talk about it… I mean… My doubts… It’s true, obviously, there’s some doubts. But let me tell you, they’re trying to address the problem; they are doing their job. You know, I’m not someone who’s very complicated at this level. Me, I take life as it comes…* **Patient 9.**

In general, there was a tacit and mutual adaptation between physicians and patients regarding the delivery of information. Physicians adapted their information to the patients’ will to be informed about treatment relevance and goals, and patients adapted their demand of information to the physicians’ propensity to communicate about these issues. Facing a patient unwilling to get involved in considerations concerning ST relevance, physicians tended to spare them from their doubts. And conversely, facing a physician who was reluctant to share with them his/her uncertainties, patients generally relied on his/her decisions without questioning their relevancies or goals. Even though most of the patients left the decisions to the doctor, most of them were also expecting clear information about their situation. Discrepancies between physician’s way to involve the patient and the expectations of the latter occurred, generating dissatisfaction and loss of trust.

*P: Me? I don’t get into it, she’s the one who decides; not me, I know nothing about it! FMN: Do you sometimes give your opinion? P: No, no, I don’t give my opinion. She doesn’t even ask for it! She told me: “we’ll do chemo”, and she chose the treatment, it’s not me. FMN: Do you feel you had clear explanations about your disease, your treatment? How do you see things? P: I’d say she’s not very communicative… FMN: And do you wish you had more time to discuss? P: That’s not the problem… One has to worm it out from her. When you ask her questions, she answers, but she’s not open, like that… FMN: What questions do you ask? P: Questions about trying to know… It’s difficult because we, as patients, we know nothing about it. So if she doesn’t want to answer, she doesn’t answer or she avoids the question. [silence] Would another doctor have given me another treatment…? I don’t know…* **Patient 19.**

Even when they chose not to share with patients their existing or predictable uncertainty about treatment relevance, physicians always included perceived patients’ preferences in their decisions. These preferences, sometimes explicit and more often tacit, were generally nominated as psychological dynamics that physicians could perceive from the patients. Most of the time, it was reflecting patients’ hope to keep the disease under control, and sometimes their weariness regarding their situation and/or treatments.

*The patient is still fighting; I think she’s willing to keep trying to find something that will improve her situation. […] I can feel it. She’s never expressed it but I can feel it.* **Physician 26, onco-hematologist.**

In several cases, physicians argued that they would continue ST even if no or little benefits were expected from it, because of the patient’s wish to continue the fight against the disease. On the grounds that it would preserve the patient’s hope, physicians spared them their reservations, and offered a treatment if any was available. If information on the risks of inefficacy or toxicity was generally delivered, these physicians remained allusive, in the best-case scenario, about the limits to come of active treatments. Sometimes, the question of therapeutic limitations and end-of-life issues was ignored until the end.

*YB: Did you discuss your doubts with the patient? MD: Doubts on what? About the treatment outcome? Or the likelihood of his general condition’s deteriorating? Well… Vaguely! Generally, one tends to say: “well, if we do nothing, we know where we’re going… Meaning often, hit the wall. If we do something, you may get a chance to avoid that. Well… avoid may sound overconfident, but at least it could improve the situation.” So that’s it, one leaves a… So for this patient, I think we discussed in those terms. And I think he answered: “If you think we can do something, let’s do it.”* **Physician 25, onco-hematologist.**

*They rarely raise the question whether one day we’ll have to stop… They’re always waiting for further treatment. In general, when we know that we will obviously do nothing more, we tend to use kind of delaying tactic… It’s a bit hypocritical, but that’s how it goes. They’re told: “we’re going to hospitalize you for artificial feeding, to recover, etc. Personally, I mostly treat ovarian cancer, and we proceed step by step. When there is no more intestinal transit, there is no more chemo, there are too many contraindications. It’s done in steps: medical imaging, then you’re hospitalized for rehydration because of your general condition… Things speak from themselves; it’s not overnight that we say “it's over”. And then, it becomes a* fait accompli *because they’ve become weaker over time. Some of them die here, but they ask few questions. It’s unspoken but it is known. We don’t force the issue by saying it’s over! We finally end up understanding each other without using words.* **Physician 8, oncologist.**

**Theme B – Second “no choice” model: giving a “last minute” choice to the patient.**

When facing uncertainty whether to continue or to stop ST, some of the physicians considered that the choice belonged to their patient.

*MD: I’m wondering if it’s a good decision. […] On the one hand, it’s difficult to take responsibility for the fact that if I renounce, I take away her chances. On the other hand, I know the dreadful prognosis of her disease. So it’s very difficult! YB: What will the decision finally rely upon, according to you? MD: I don’t know yet. Maybe on what she tells me. Her wish, because I’m going to try to speak to her as honestly as possible, not to influence her response… Because it’s easy to influence a response.* **Physician 29, oncologist.**

Physicians discussed their influence on patients’ preferences in some interviews. They generally referred to the dominant model described above, in which patients tended to rely on the physician’s decision, agreeing to proposed therapies and usually understanding the necessity to discontinue or stop specific ST when they couldn’t be continued.

However, the nature of the choice left to patients when they had to decide between continuing or withholding of ST, was not discussed by physicians in the interviews. Yet, several patients expressed the difficulty this could represent for them.

*If* I *have to make decisions? I don’t know if I could, personally… I’d have the feeling I chose the wrong thing…* **Patient 26.**

Another patient implied that between, on the one hand, a therapeutic possibility inevitably associated with hope – as small as it may be – and on the other hand, therapeutic abstention, meaning the reality of the coming death, there actually was no choice.

*Anyway, either we do nothing, or we do something that has a little chance! One must take a chance, somehow… Personally, I am pragmatic: It’s yes, it’s no, we’ll make it or we won’t… But if we don’t try…* **Patient 9**

In many situations, physicians seemed to use their influence to convince patients that further treatments were justified until they began to doubt whether it would benefit them, generally as cancer became far advanced. Then, as the disease became unresponsive to treatments and/or the patients’ general conditions worsened, and predicting more or less consciously that death may be imminent, physicians started to either convince them of the opposite, or to give them the choice to continue or stop ST.

This “no choice” situation, before which some physicians placed their patients, hampered an “informed” decision on the part of the latter, particularly since end-of-life issues had generally not been previously discussed. Except for two patients suffering from hematological malignancies, for whom a curative goal was still expected in spite of a questioning on pursuing or withholding ST because of morbidity, all the physicians expressed that they told the patients about the incurable nature of their cancer. However, fewer physicians expressed having shared with their patients about the on-going questioning (18 of the 29 physicians, and 12 of the 21 whose patients could be interviewed). Even fewer physicians expressed having earlier discussed with their patients (not including the on-going questioning) that ST might possibly be limited or stopped at a certain point of the disease trajectory (7 and 4 respectively, of the two groups described above). Finally, only four physicians mentioned that they had discussed about death or end-of-life preferences with their patients. Two of these conversations had been initiated by patients themselves.

From the patients’ point of view, incurability was generally understood, and they were often aware of disease resistance to ST. However, half of the patients expressed unrealistic expectations from ST compared to those of their physicians. Only a third seemed aware of their physician’s questioning on therapeutic limitations. All the patients wanted ST to be continued at the time of the interview. Among them, four expressed ambivalence due to apprehension of ST morbidity and/or weariness toward active care of their disease. Several patients faced difficulties expressing their fears or their doubts with their physicians. Sharing these was sometime discouraged by physicians, and thus they were unable to be taken into account of in the questioning about the goals of care.

*I wish they could find a treatment that’s not too difficult and that works, that’s it! […] I’ve already told them twice: “I’m fed up… Anyway, I’ll stop everything… we’ll see what happens!” – “Oh no, we can’t do that!” So, I said: “Yes, I know…” – “No, no, I’m not allowed”, he said, as if to say: “even if you wished…” I talked about it twice […] not to provoke, but because I’m fed up, you know, it just isn’t working!”* **Patient 26.**

*P: Yes I trust them, but still, I have some questions! FMN: Yes? Which ones, for example? P: Is my 3* ^*rd*^ *chemo going to do the same thing [pulmonary toxicity] or are they going to change it again? That’s it… Because I don’t want to be hospitalized for 10 days, every 21 days… FMN: Do you get answers to that? P: Not really… Because I have the feeling they don’t really know…, that’s basically it…* **Patient 4.**

*I asked again this question, today: is it worthwhile? As he [the physician] says, there’s always hope, since we haven’t tried every treatment… But when I see myself like this… It’s hard to imagine that my life will last a long time. […] I think that we cling to any little thing, and if something exists, we try, even if sometimes we just want to give up… Even so, it’s hard to just give up.* **Patient 16.**

**Theme C - Early engagement in shared reflections and dialogue about uncertainties and limits with patients, proxies and teams: complexity and subtlety of shared decision-making.**

As long as the dynamics of disease control through ST was at stake, conversations between physicians and patients were dominated by the medical and technical issues of treatments and were driven by the need to maintain hope based on treatments. Many physicians underlined contradictions in advanced cancer patients’ care: supporting patient autonomy while respecting her/his limits and psychological integrity; not neglecting patients’ fears or needs, and anticipating end-of-life issues while active treatments were still justified and made it possible to easily avoid these topics; sharing their uncertainties and treatment limits with patients, when they expected reassurance and hope… These tensions were considered as wearisome by most physicians. However, some of them, belonging to the same investigation centre, considered they were challenging communication issues, implying anticipation and continuous adjustment.

*Before deciding to begin chemotherapy, we discussed a lot about the advantages and the disadvantages [with the patient] … So I explained at lenght the risk-benefit balance without excessive valuation of chemotherapy, and really, above all, taking into account Ms. B’s view, and explaining the issues. So it wasn’t “Listen : here are the response rates, here is the higher survival rate with treatment, versus we do nothing and we do palliative care”… it’s not as clear-cut as that, this kind of discussion doesn’t help. No, I just involved her in what we can do, what we cannot… the importance of comfort care in parallel… She was aware of these issues very early.* **Physician 12, oncologist.**

These physicians frequently mentioned that anticipating uncertainties and the limits of treatment in the discussions with patients and with the care team was a major issue of care. They considered that, by working early, progressively and cautiously on this issue with patients, they allowed the emergence of benchmarks, or thresholds beyond which pursuing an active stance against the disease by means of ST would become inadequate. This encouraged mutual agreement based on the respect of values, priorities or the quality of life conceptions of the patient. In their opinion, achieving this required personal thoroughness and rigor, as well as interdisciplinary team, to comprehensively take into account the patient within his family system.

*Efforts in communication are needed: the way of going about things, verbal and non-verbal communication, taking into account others around who may have influence, time management – one’s own timing, and respecting the pace of the person in front of us, what he says to other caregivers that he doesn’t say to us. […] In the end, one could find it rather reassuring to apply guidelines. But guidelines, either for undertaking or desisting treatments, won’t solve the question of how and when to discuss either with the patient.* **Physician 11, oncologist.**

*Yes, [these multidisciplinary meetings] help. They bring me other points of view, especially those of the caregivers who interact with the patients at different moments of the day, not necessarily at the same time as I do. It gives me the opportunity to know what happened over the last few days, if I haven’t seen the patient every day, and to see if my feeling is the same as theirs, and how they consider things. So it is an opportunity to have different notions in time and other points of views.* **Physician 12, oncologist.**

Conversely, interviews with physicians and patients showed that a shared questioning about therapeutic limitations and end-of-life issues was difficult and sometimes impossible to initiate when this was “up against the wall”. For both of them, stopping ST was associated with resignation to the coming death. When physicians faced the clinical impossibility to continue ST and looked forward to discuss these issues with patients (often for the first time), either psychological defence mechanisms or anguish about death seemed to hamper both physicians’ and patients’ ability to value a “life-after-ST”, and to identify issues making this “after” not just a hopeless and morbid waiting time. These psychological defence mechanisms were identified in many of the physician’s and patient’s words:

*I told him: “We’re not doing chemo tomorrow.” And then, he looked at me with horrified eyes, and told me: “That’s not possible, if we don’t do chemo, I won’t be alright!” So, I said “but you can see that you’re not alright, now, despite the chemo!” So he was a little… he hesitated, and then he answered: “No, no, I want to do it! I can’t just not do it!”. That’s it, for him it wasn’t even conceivable. Stopping treatment wasn’t conceivable. I told him: “Listen, I can… If you want we can do it, but it’s the last one!” Actually, I cheated a bit; I gave in on that point, telling that we wouldn’t do anymore [chemotherapy]. It was a bit stupid, because he could have said the same the week after… and what would I have done?* **Physician 18, oncologist.**

*P: I’d like to continue, but as long as… First of all, I’ve not defecated for 10 days, and there is still no sign… They’re trying their best to liberate the bowels, but that’s it, that’s where we’re at. YB: Is this the reason why chemotherapy is not being continued for the moment? P: No, we’re still doing it… But you see, I was told it would be long. For the previous time, it was already long, because I didn’t defecate for 14 days… YB: Would you need more discussion or explanations from your doctor about the current situation? P: [whispering] There’s no point… YB: Why? P: Because I very much hope, so there’s no need to… to know too much…* **Patient 20.**

### Individual and systemic barriers to a shared questioning

#### Individual barriers

Barriers related to individual (caregivers’, patients’ or proxies’) limits appeared to hinder a timely and sensitive sharing about uncertainties and treatment limits, which in turn influenced medical decision. It seemed these individual limits were related to the varying capacities each to cope with disappointed hopes, which were at first possible, then likely, and finally certain: hope to be cured, to live longer, to release suffering for patients and their families; hope to cure, to prolong life, to provide answers and solutions for the physicians.

*YB: Is this feeling of giving up strong, when treatments are stopped? MD: Yes, I feel it intensely, because I was taught to heal people and not the other way round! We’re not here to tell… We do it every day, but it’s rarely easy.* **Physician 1, hematologist.**

*It’s a bit like giving up, since as soon as we don’t treat the disease anymore, we know very well that it will evolve towards a tragic end. Maybe it’s a kind of personal failure statement too. One thinks “that’s it, I can’t stabilize or slow the progression of the disease!” But most of all, it’s the feeling towards the patient… one feels that when one decides to stop, it’s a kind of death sentence… That’s exactly what it is.* **Physician 26, onco-hematologist.**

Patients and physicians facing a possible discontinuation of ST seemed to experience individual suffering, consisting of fear of death, anguish, and feelings of giving up, failure or guilt. Furthermore, the suffering of each was likely to be mutually reinforced. Thus, frequent avoidance of shared questioning about uncertainties, helplessness and end-of-life issues seemed to be fostered by a mutually reinforced refusal to “give up”, driven by a mutually reinforced avoidance of suffering.

In contrast, some physicians considered that acknowledging, accepting, and possibly sharing their emotional involvement in these situations were part of their professional responsibilities, so that their decisions could remain centred on the patient’s interest.

*One has to acknowledge that it’s one’s job, and that this [end-of-life decision] is going to happen. But it’s true that we get more attached to some patients, and that’s natural […] I think that what matters most is that we do our best, trying to keep an eye inside on what relationship we may have with patients so that decision remains fair. We can’t always keep control, far from it, and that’s not the goal either. Not exposing oneself is also important to avoid getting into feelings of failure, etc. I’m quite aware of this, and I believe that I’m quite conscious about how I get involved, including on the emotional side. So it happens that, sometimes, I let it go, and fortunately we have a psychologist who helps to get it off one’s chest, when we find it hard.* **Physician 10, oncologist.**

#### Systemic barriers

In addition to individual barriers, physicians pointed out systemic and/or institutional barriers that contributed to the hindrance of in-depth questioning regarding uncertainties and limits with the teams and the patients. They named several obstacles: lack of time; lack of appreciation of deliberative and reflexive time; lack of appreciation of the information and support time with patients and families; lack of communication and ethical reflection training; and lack of psychological support for caregivers.

*MD: We do out-patient consultations early in the morning, we hurry up to go see [hospitalized patients] or determine a goal because our time is limited. And sometimes we don’t have time… that’s it, our own time. YB: How does this influence your decisions? MD: In the sense that we don’t take the time to listen to what the patient has to say, nor the time to explain. […] We don’t take the time to let the patient set his/her pace, and say things…* **Physician 21, oncologist.**

*Is it easier with experience? I’d say no, it’s less and less easy, because we’re not psychologically supported despite the difficult situations we may face, the bad news we have to give… We might deliver very good news, and 10 s later, we might announce death. So these sudden turnaround situations for hospitalized people or out-patients are difficult because we don’t have an outlet; we don’t have anybody to speak to about it with.* **Physician 1, hematologist.**

*We don’t have the words, we were not trained for that. So we use everyday words without knowing if we’re… if they’re the right words, if they’re fair according to the psychological context. We were not trained for that… Personally I wasn’t trained. So we blurt it straight out, and we hurt…* **Physician 25, onco-hematologist.**

## Discussion

This study reports for the first time, to our knowledge, (1) a prospective measurement of the prevalence of the questioning about the relevance of ST in hospitalized advanced cancer patients and (2) a mirror prospective analysis of physicians’ and patients’ points of views concerning the decision process. These questionings concerned nearly 13 % of hospitalized patients. They were rare in the day or week units, and we observed a heterogeneous distribution among the different centres.

The timings of these questionings were variable since they could be initiated before any ST was started as well as after several treatment lines, when no further treatment was feasible. However, most of them occurred as physicians estimated a life expectancy comprised between 15 days and 3 months. This timing seemed to rely on clinical factors: cancer type and extension, its clinical impact, the clinical background, previous and/or expected treatment efficacy and tolerance, or availability of further treatments. Other factors emerged from our analysis of physicians’ and patients’ interviews: (1) individual factors related to their respective capacity and propensity to go into and share a questioning about knowledge limits (uncertainty), controlling limits (helplessness), and life limits (death); (2) factors related to unit sub-cultures; and thirdly, factors related to institutional constraints. These results bring a deeper understanding to the subjective determinants of the prescription of chemotherapy already reported in other studies [16].

Appropriate timing for initiating shared questioning with patients about treatment limitations (i.e. end-of-life issues) has been widely debated in the last few years. Our results show that physicians may adopt various postures, depending on patients’ limits as well as on theirs. A mutual adaptation seems to occur, explaining that a physician and a patient could be satisfied with the care while end-of-life issues were never discussed, whereas others had engaged such discussions earlier and beneficially. However, discrepancies occurred, raising ethical issues. Such ethical issues have been recently discussed on the basis of a qualitative analysis of physicians’ and nurses’ interviews in Germany [26]. Results reported by Temel et al. showed that quality of life, psychological consequences and length of life were improved in metastatic pulmonary cancer patients as early palliative care, in collaboration with oncological care, notably focused on patients’ understanding of prognosis [10, 27, 28]. Several studies revealed that many patients were able to keep hope alive while acknowledging the terminal stage of their disease, and that most of them were expecting honest and precise information from their physicians, given with empathy and sensitivity [17, 29–31]. However, these discussions are difficult, and may be harmful for patients in terms of quality of life and the psychological impact [32]. Thus, they have to remain careful, tailored to each patient, and contained in a professional framework where communication training is essential [33, 34].

Indeed, results presented in this study show the complexity and the subtlety of the patients’ involvement in the decision process. We report the modalities of the questioning, and even more of its sharing, that were often sacrificed to a relational dynamic, mutually reinforced, focused on hope and “not giving up”. These results are consistent with the findings of Buiting et al. [18], and allow a better understanding of the Western countries’ concern, that terminal cancer care is increasingly aggressive [9, 13–15, 35, 36]. We describe a dominant model where physicians and patients faced a “no choice” decision, when physicians were striving to resolve the dilemma between beneficence and non-maleficence by the means of two different ways. Firstly, by means of a technical answer or a “wait-and-see” attitude–letting the evolution of the situation decide by itself. This way of responding to uncertainty seemed favoured by the fact described in other studies, that some patients were reluctant to be involved and some physicians to involve them in the decision process [19, 37–39]. And secondly, by deciding to leave the choice between pursuing or discontinuation of ST to the patient. This attitude, described elsewhere in the literature, seems to match a minority of patients’ preferences only [40–42], and is likely to put them in a difficult “no choice” position, where they have to choose between inevitability of the death and potentially futile or harmful (but “hope supporting”) treatment. In the other decision-making model that we describe, the physicians initiated early shared reflections and dialogue about uncertainties and limits with patients, proxies and teams, allowing the emergence of individual benchmarks, or thresholds making it possible to guide the decision about the appropriate role of ST in a given situation. The relevance of such an approach in clarifying specific goals of care for each patient was thoughtfully discussed by Emanuel et al. [43]. The propensity of physicians to use this second way of dealing with uncertainty seems to depend on how they consider their professional role, and if, in addition to the responsibility of being competent bio-clinicians, a psycho-social role is assumed: helping patients and families to cope with advanced or terminal disease [44]. This posture is likely to be consistent with erratic trajectories of advanced cancer patients, in which therapeutic options are multiple and changing, as they were described by Kort et al. [20].

The main obstacle to implementing this model seems to be related in this study, as in the literature, to a systemic and cultural relational dynamic into which, through ST, the need for hope is converted into fighting the disease, and dealing with suffering is partly avoided as long as ST remain available [18, 45, 46]. Mostly unconscious and mutually reinforced defense mechanisms are at stake. They may protect each other from experiencing overwhelming distress in these situations. But they also may divert attention and questionings from fundamental issues other than disease control in advanced cancer patients, such as: appropriate pain and symptom management; avoidance of a prolongation of life in unacceptable conditions because of unrealistic hope; preservation of self-determination; bereavement support; and mediation in patient/family relationships [47]. Multidisciplinarity [48] and proxies’ integration [49] in end-of-life discussions are essential to personalizing the care of advanced cancer patients.

The interpretation of the results reported here must take some limitations into account. First, the saturation of concepts was applied and reached in a second step, as we chose to conduct all the physicians’ interviews, and a maximum of the eligible patients’ interviews. However, even if patient sampling was not guided by the saturation of concepts, we tested the validity of the emerging models through a saturation of concepts *a posteriori* and repeated cross analysis by our multidisciplinary research team. Secondly, having included hematology patients could be considered as a limitation, since a potential curative goal remained for some of them, probably reinforcing the not-giving up attitude observed elsewhere [50–52]. Nevertheless, the shared questioning model that we developed still seemed to be valuable even when concurrent with justified active treatment, suggesting that it may be valid in both solid cancer and hematological malignancies. Finally, the frequency of the questioning is likely to underestimate the reality, as it was recorded during one week only and excluded out-patient consultations.

## Conclusion

Personalized medicine is central to current public health issues in economically developed countries. In oncology and hematology, the term covers different realities, from progress in targeted therapies to improvements in coordination and in the networking of caregivers [6, 53]. Our study highlights essential determinants of the decision-making process of the pursuit, limitation or discontinuation of ST in advanced cancer patients and the personal and inter-personal ability to question and share about uncertainty and limits in an ethical approach. Improvements have to be made in the acknowledgement and the implementation of such an approach, without which personalized medicine remains different from a patient centred medicine.

Efforts still need to be developed and intensified in several fields, including: initial and ongoing training of caregivers working with cancer patients on ethical, communicational and end-of-life issues; developing a caring environment allowing the implementation of a real transdisciplinary approach and decision-making process; giving value to deliberative and reflexive time, regarding them as health care interventions integrated in the financing system of care units; encouraging patients and health care users to get information from and discuss goals of care with their physician. Research on caring or experiencing advanced cancer situations and end-of-life should be developed and favoured, as important gaps remain in many fields, such as symptoms management, understanding of seriously ill patients’ and their caregivers’ needs depending on their cultural background, decision process, or physicians’ and patients’ interactions in the end-of-life context [54].

### Availability of data and materials

All data sets on which the conclusions of the paper rely are available on request to the corresponding author.

## Abbreviations

QST: questioning about the pursuit, the limitation or the withholding of specific therapies
ST: specific therapies
